# Cell-Assisted Lipotransfer in Breast Augmentation Surgery: Clinical Outcomes and Considerations for Future Research

**DOI:** 10.7759/cureus.22763

**Published:** 2022-03-02

**Authors:** Dimitrios Asimakopoulos, John M Anastasatos

**Affiliations:** 1 Plastic and Reconstructive Surgery, University of Cambridge School of Clinical Medicine, Cambridge, GBR; 2 Plastic and Reconstructive Surgery, Providence Cedars-Sinai Tarzana Medical Center, Los Angeles, USA

**Keywords:** growth factors, cell-assisted lipotransfer, stromal vascular fraction, lipoaspirate, adipose-derived stem cells (adsc), autologous fat transfer

## Abstract

Autologous fat transfer is a widely used surgical technique, chosen by numerous plastic surgeons for breast augmentation surgery. This technique is based on three steps: 1. harvesting of the lipoaspirate from the patient, 2. centrifugation and removal of the top, oily, layer, and 3. implantation in the patient’s breast(s). It has been associated with various complications, including post-surgical fat resorption, as measured quantitatively with MRI, CT, and other 3D-quantification systems.

Adipose-derived stem cells have been explored as a means of addressing fat resorption. They can be separated from the lipoaspirate following centrifugation, and enzymatically purified from unwanted debris, with collagenase, forming the stromal vascular fraction. The stromal vascular fraction is then recombined with the graft volume prior to implantation. This novel technique, referred to as “cell-assisted lipotransfer”, has shown promising results in terms of reducing fat resorption. These results are due to the pro-angiogenic and pro-adipogenic ability of the stem cells, which allow the graft to address the conditions of ischemia more effectively than autologous fat transfer.

The aim of this review is to explore the ways in which cell-assisted lipotransfer is different from the autologous fat transfer, as well as how and why adipose-derived stem cells may contribute towards limiting fat resorption. The immunological background of these cells is discussed in detail, while grounds for further development are discussed, by means of the administration of external growth factors, which could, potentially, maximize outcomes, while limiting complications.

## Introduction and background

Autologous fat transfer and cell-assisted lipotransfer

Breast Augmentation Surgery

Breast augmentation is among the leading types of cosmetic surgical procedures in the United States, with 299,715 procedures in 2019 and 193,073 in 2020 [1]. Worldwide, it accounts for 1,795,551 procedures in 2019, according to statistics by the International Society of Plastic Surgery (ISAPS), constituting the leading type of cosmetic surgery [2]. For many years, the conventional approach towards breast augmentation has been the use of implants of varying composition, including silicone gel, saline-filled, double-lumen, textured surface, and anatomic and round-shaped implants, aimed at shaping the volume and contour of the breasts [3,4]. Mammary implants present with various complications, associated with the surgical nature of the intervention, including haematomas, infection, loss of areolar sensation and pain, and implant-related complications, including capsular contracture, rupture, and displacement [3,4]. 

Autologous Fat Transfer (AFT)

AFT is the technique of choice for lipotransfer in breast augmentation, with the first efforts as early as the late 19th century [5]. It takes advantage of the fact that adipose tissue is a malleable type of human tissue, which can be extracted in a minimally invasive way from numerous body areas. This technique is divided into 3 stages: 1. harvesting of the lipoaspirate, 2. processing, and 3. implantation [5]. The greatest challenge during these steps, particularly centrifugation, is the minimisation of injury to the adipose cells, given that they will be transferred to a nutrient-poor and less well-vascularised environment [5]. Harvesting of the fat can be performed through manual aspiration, or through low-pressure vacuum liposuction [6]. Processing consists of purifying and centrifuging the fat graft with care, to avoid exposure to air, which could compromise its survival [5,6]. Lastly, centrifugation is aimed at separating the middle and lower phases of the lipoaspirate from the superior phase, which contains proteases, lipases, and lipids that may compromise the graft, given their enzymatic and pro-inflammatory capacity [5]. AFT is currently used to correct breast deformities, particularly following ageing, trauma, congenital deformities, cancer excision surgery, and radiotherapy [4,5,7]. This is the starting point of this review, which assesses to what extent different techniques have been developed, with more promising results.

Cell-Assisted Lipotransfer (CAL)

Taking AFT a step further, adipose-derived stem cells (ADSCs) can be extracted from the patient’s lipoaspirate, after harvesting. The usefulness of ADSCs in lipotransfer is explored in detail throughout this review, in the context of their pro-angiogenic and pro-adipogenic profile, which has been shown to improve fat graft viability following implantation in the patient. This novel technique has been shown to be effective for volume increase and breast contour reshaping [8-10], without the invasive nature of breast implantation [4]. Multiple studies have highlighted the promising results of CAL. However, current literature on CAL lacks standardised procedural methodology, while the immunological mechanisms of ADSCs are yet to be fully characterised [11]. 

It should be noted that research into stem cell therapies has been performed in various fields, such as orthopaedic surgery, for purposes of enhancing tissue repair [12]. The widespread applications of these therapies stress the importance of exploring the use of stem cells in breast surgery, aiming to optimise patient safety and outcomes [12]. The U.S. Food & Drug Administration has been regulating the use of such therapies closely, given the risks to patient health, as a result of the use of unproven or unapproved protocols [13]. 

## Review

Limitations of autologous fat transfer and need for a novel technique

AFT allows for the preservation of natural post-surgical aesthetics of the breast, particularly in the context of reconstructive breast surgery following mastectomy [14,15]. However, it has been associated with increased fat resorption, which varies between 25-75%, primarily due to fat apoptosis, necrosis, and liquefaction [4-6,16]. This wide range of fat resorption has been reported by multiple authors and hints at the fact that a range of factors might influence its extent, without, up to this point, having a clear understanding of their individual importance. Such factors may include the volume of the graft implanted and methodological variations in different techniques [17]. 

Fat resorption is the result of both cellular apoptosis, and necrosis, with adipocytes being particularly susceptible to death [18]. Resorption is observed primarily in the centre of the volume of the lipoaspirate since post-surgical revascularisation commences at the periphery and progresses inwards. Eto et al. explain that fat grafts generally consist of three concentric layers of fat, the innermost (“necrotic zone”) being the one with the highest proportion of adipocyte and ADSC death, and the outermost (“surviving zone”) being the one with the highest survival of adipocytes [18]. Survival in the middle layer (“regenerative zone”) depends heavily on the hypoxic conditions, and the extent of vascularisation from surrounding tissues [18]. A potential way to address resorption is to transfer fat in small volumes, therefore increasing the surface-to-volume ratio of the aspirate, to minimise the proportion of fat that is not well vascularised [7]. 

Another complication that may occur in AFT, similar to mammary implants, although not necessarily as frequently, is an infection of the operating site, which can range from a minor infection to the formation of an abscess that requires further intervention [6].

Cell-assisted lipotransfer as a novel technique in breast augmentation

Extraction of ADSCs

CAL has been introduced in the past few years as a technique that shows promising results in terms of optimising the survival of the fat graft, based on the principles of AFT [16]. The lipoaspirate used in AFT contains ADSCs, which can be extracted at much higher numbers and with a less invasive technique than other types of stem cells, such as bone-marrow-derived stem cells [19,20]. Targeting ADSCs has shown promising results in terms of optimising fat graft survival in breast augmentation. 

Harvesting and Processing of the Stromal Vascular Fraction (SVF)

ADSCs are not extracted with the intention of multiplying them in vitro, but rather to concentrate them prior to reuniting them with the lipoaspirate for the purposes of implantation [19,21]. This can be achieved either through enzymatic or mechanical processing of the fat graft. Both these ways of processing the graft will generate the SVF, which is a network of cells, primarily endothelial cells and pericytes, and an extracellular matrix, containing numerous ADSCs [8].

**Figure 1:**
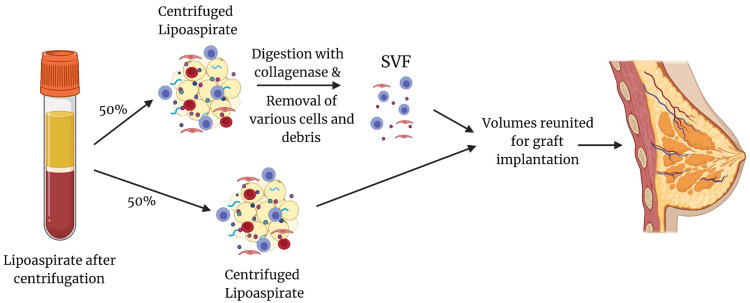
Generation of the stromal vascular fraction through enzymatic processing with collagenase This image has been created with BioRender.com, and licensed for publication [9,19,20]

In enzymatic processing of the fat graft, following centrifugation, some of the collected volumes are allocated to the lipoaspirate, while the rest is allocated towards the generation of the SVF, as illustrated in Figure 1. The volume that is allocated towards the SVF is subsequently digested in a solution of collagenase and then undergoes further cycles of centrifugation. In mechanical processing of the fat graft, the SVF is generated by centrifugation and filtering through a -0.2 μm-filter [8]. It should be noted that even after processing, the SVF remains a heterogenous population of cells, containing haematopoietic stem cells, vascular endothelial cells, and pericytes, but the removal of other cells and extracellular material means that ADSCs become more concentrated, compared to before treatment. [4,22-25]. Furthermore, ADSCs have been suggested to interact with other cell types in the SVF, therefore prolonging their survival in the hypoxic environment of the graft, as opposed to a population only made of ADSCs [9]. The presence of additional cell populations in the SVF suggests that if an ADSC-only population were to be used instead of an SVF population, ADSC cell expansion would potentially have to take place in order to achieve the same volume; cell expansion may entail further legal concerns, as explored below.

Centrifugation has been shown to concentrate ADSCs by as much as 43%, while increasing the survival rate of the graft, up to 3,000 revolutions per minute. Beyond this centrifugation rate, significant cellular damage has been reported by various authors [9,26]. The clinical advantage of CAL over AFT lies in, 1. the ability of ADSCs to endure longer periods of hypoxia, particularly since the fat graft is not vascularised when transferred to the breasts, and 2. the pro-angiogenic and pro-adipogenic ability of ADSCs, supporting the viability of the fat graft post-implantation [4,27]. 

A potential question arising from Figure 1 is why various authors suggest using only 50% of the lipoaspirate to generate the SVF, rather than a higher percentage, up to 100%. The answer lies in how SVF is produced, through enzymatic or mechanical processing. Following this processing, the product does not contain adipocytes. Therefore, it cannot constitute the fat graft on its own, without mixing with adipocytes from the centrifuged lipoaspirate, as shown in Figure 1. This question could be further extended to investigate how changing the percentage allocated to SVF and the lipoaspirate alters fat graft survival, to find the optimal proportions of the two volumes.

Finally, it should be emphasized that in the United States, CAL is considered as “more than minimal manipulation”, due to “altering the original relevant characteristics” of adipose tissue, as per the FDA’s 2014 guidance, and hence regulated under section 351 of the Public Health Service (PHS) act as a drug [20,28]. Furthermore, the SVF used in CAL is distinguished from AFT, which is considered “minor handling” [20]. 

Clinical outcomes of cell-assisted lipotransfer

Radiographic Analysis of Outcomes

Quantitative imaging methods, such as MRI, CT, or other 3D systems, allow for the volumetric analysis of the outcomes in terms of fat graft survival. In a systematic review and meta-analysis, Laloze et al. suggest increased survival rates following CAL (61%), compared to non-CAL breast augmentation procedures (45%), as compared to 69% vs. 51%, respectively, in facial filling [29]. 

In another paper, Zhou et al. have found CAL to increase fat survival rates from 45%, in AFT, to 60% in breast grafting. In the same study, a bigger enhancement in survival rate was observed in facial grafting, from 52% to 71% [27]. Facial grafting is generally associated with lower complication rates across multiple studies [30]. Support towards CAL vs centrifuged fat has been provided by other authors, as illustrated in Table 1, which compares CAL vs. AFT, as discussed by four systematic reviews and meta-analyses.

**Table 1:** List of systematic reviews and meta-analyses, with their respective data on fat graft survival and complication rates, comparing CAL versus. AFT CAL: cell-assisted lipotransfer, AFT: autologous fat transfer Zhou et al. [27]; Laloze et al. [29]; Chen et al. [30]; Li and Chen [34]

Study	CAL fat grafting		AFT		Fat survival ratio (CAL vs. AFT)	Complication ratio
	Fat survival rate	Comp/on rate	Fat survival rate	Comp/on rate		
Zhou et al.	60%	12.6%	45%	11.1%	1.33	1.14
Laloze et al.	61%	12.1%	45%	3.8%	1.36	3.18
Chen et al.	69%	12.8%	51%	6.1%	1.35	2.10
Li and Chen	Did not report individual fat survival rates and complication rates				1.79	1.34

Use of Different Isolation Systems for SVF

There exist various SVF isolation systems, such as Celution (by Cytori Therapeutics, Inc.), Medikhan (by Medi-Khan Inc.), Fatstem (by Fatstem CORIOS Soc. Coop) and Mystem (by Mystem evo Bi-Medica) [8,31]. Of these systems, the first two utilise enzymatic processing, while the last two use mechanical processing to generate the SVF [8]. The presence of procedural differences among these isolation systems complicates the comparison of CAL with AFT [8,21,32,33]. Furthermore, the above systems perform automatic isolation, but SVF can also be isolated manually. Numerous authors have investigated potential differences in fat survival rates, as a result of automatic SVF isolation systems vs. manual systems, without reporting any significant differences to this date [27,29,30,34]. Table 2 compares the survival rate of the fat graft, by using different automatic SVF isolation systems, as compared with AFT grafting. An optimal number of cells in the SVF, prior to grafting, has not been determined yet, given the methodological differences in individual methods of isolation [27,29,30].

**Table 2:** Comparison of fat survival rates across various automatic SVF isolation systems versus AFT SVF: stromal vascular fraction, AFT: autologous fat transfer Gentile et al. [37]; Gentile et al. [8]

Study	CAL system fat survival rate	Autologous fat grafting survival rate	Ratio of CAL vs. AFT approach
Gentile et al.	63% (Celution)	39%	1.62
Gentile et al.	52% (Fatstem)		1.33
	43% (Mystem)		1.10
	39% (Medikhan)		1.00

Histological Evaluation of Outcomes

Apart from radiographic methods of evaluation, Kølle et al. performed a randomised, placebo-control trial and analysed histological data of ADSC-enriched vs control grafts. They concluded that there was a statistically significant increase in the distribution of both adipose (84.3% vs 67.0%) and newly formed connective tissue (5.3% vs. 0.5%), in ADSC-enriched tissue, compared to the control group, as well as reduced distribution of necrosis (4.6% vs. 16.1%) [35]. Additional evidence has been provided by Gentile et al., who performed histological analysis of 46 patients’ fat grafts prior to implantation for breast augmentation, showing elevated concentrations of ADSCs in the group treated with SVF, compared to the control group [36].

Limitations of cell-assisted lipotransfer 

The improved clinical outcomes in terms of graft survival come at the cost of certain complications. Table 1, above, shows higher complication rates in CAL versus AFT procedures. It also shows the significant variance in complication rates, and as such, it would be interesting to further investigate why this is the case. A potential explanation for the discrepancies in complication rates is the use of different preparation protocols, given that CAL does not have a standardised technique. Similar to AFT, some of the complications listed in the “Introduction to AFT and CAL” section with regards to mammary implants, such as infection and the formation of a haematoma, also apply to CAL, due to procedural similarities. Crucially, the potentially increased risk of carcinogenesis is evaluated further in the section on "External administration of growth factors".

Formation of Cysts

Laloze et al., Chen et al., and Zhou et al. point out that the primary complication associated with CAL is the formation of cysts [27,29,30]. Automatic isolation of SVF correlates with higher rates of cyst formation (6.9%) than manual isolation (1.6%), as per Laloze et al. [29], and 11.1% versus 2.5%, respectively, as per Zhou et al. [27]. Therefore, it seems that the method of automatic isolation of SVF may in part explain the increased complication rates of CAL, as compared with AFT.

The Ratio of Surface Area to Volume

Higher, and statistically significant, CAL-related complications have been reported by Laloze et al. when graft volumes were ≥100 mL as opposed to <100 mL. Laloze et al. further explain they did not find a statistically significant difference in complication rates at higher volumes in non-CAL techniques [29]. This may be an additional explanation for higher complication rates in CAL vs AFT. This finding is in parallel with the paper of Eto et al. [18] with regards to the three concentric layers of the fat graft, highlighting the importance of the ratio of surface area to volume [38].

It might prove beneficial to compare a one-time CAL fat transfer, with multiple fat transfers over several procedures, with regards to results and complications. The limitations of doing so involve operating on a patient on multiple occasions, if the fat graft is not inserted in full at once, as well as the storage of the lipoaspirate in between procedures, which could be achieved by means of cryopreservation [39].

Calcification

As reported by various authors [4,27,34], calcification constitutes a commonly reported finding observed after CAL procedures, due to inflammation-mediated dystrophy. However, this is not unique to CAL, as calcification can also occur with conventional AFT [4,6,40]. 

Other Considerations

CAL is a more recently developed technique than AFT, and as such, additional concerns can be pointed out. Breast augmentation procedures involving CAL require more operating time than AFT, by 90-150 minutes, as SVF isolation alone requires approximately 90 minutes. Furthermore, breast augmentation surgery is already expensive costing approximately $8,000 for the procedure and ancillary support [41], so adding CAL to the clinical plan would result in further increased costs for patients and providers [9,32].

Patient-reported outcomes

Much of the available bibliography highlights the need for reporting of patient-reported outcomes, without subsequently offering in-depth analyses of these outcomes, and justifying the criteria on which such evaluations were based [4]. 

Pérez-Cano et al. evaluated patient and clinician satisfaction using the LENT-SOMA and Quality of Life scales, and Clough’s classification system [10]. They reported satisfaction with the appearance of the breasts in 45 out of 67 patients (67%) and 58 out of 67 clinicians (87%), at 12 months. With regards to the overall treatment process, they reported satisfaction in 50 out of 67 patients (75%) and 57 out of 67 clinicians (85%) [4,10,42].

The results of this study show a discrepancy in patient and clinician-reported outcomes across reported outcomes. This could be due to a variety of reasons, including patient-clinician miscommunication, or differences in the perception of the outcome of the surgery by clinicians and their patients. It seems necessary to further explore the potential causes of patient dissatisfaction, including expected aesthetic outcomes.

Cell markers and cytokine profile of cell-assisted lipotransfer

Angiogenesis is the formation of blood vessels, mediated by the differentiation of precursor stem cells into endothelial cells and fibroblasts, through the release of various growth factors, targeted towards pre-existing vascular structures. Essential growth factors include vascular endothelial growth factor (VEGF), platelet-derived growth factor (PDGF), fibroblast growth factor (FGF), and transforming growth factor-β (TGF-β) [38], while matrix metalloproteinases also play a role in vascular remodelling [43,44].

**Figure 2:**
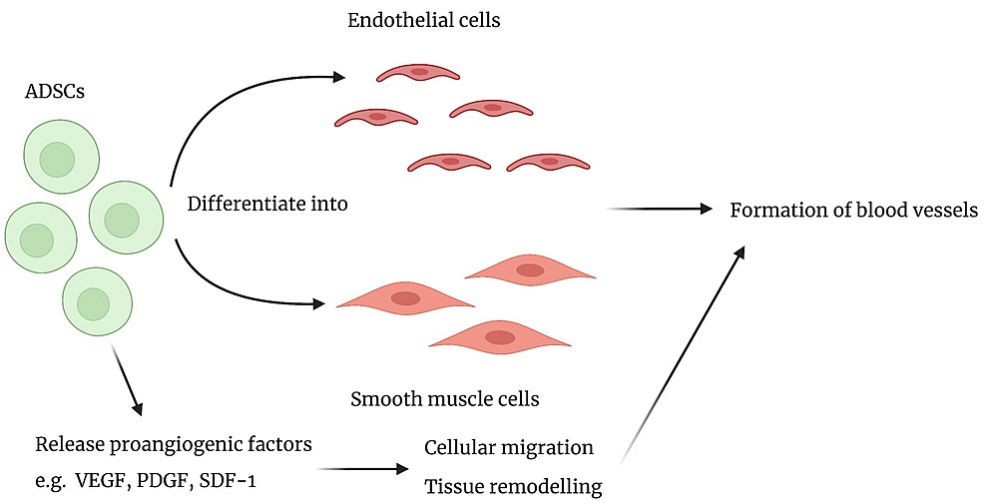
Schematic of the pro-angiogenic profile of ADSCs ADSCs: adipose-derived stem cells; Figure 2 has been created with BioRender.com, and licensed for publication [47,48]

ADSCs are progenitors of vascular endothelial cells and adipocytes, releasing some of the above growth factors, including VEGF, TGF-β, hepatocyte growth factor (HGF), and stromal cell-derived factor-1 (SDF-1), in a paracrine manner, in response to conditions of hypoxia, while they are more efficient at proliferating than bone-marrow-derived stem cells [5,45-47]. Hypoxic conditions could occur due to insufficient vascularity when the fat graft is implanted in the patient’s breasts [16,25,27]. Studies have shown that the levels of VEGF expressed in ADSCs can reach 5-fold of the baseline under hypoxic conditions. This pro-angiogenic profile enables ADSCs to differentiate into endothelial and smooth muscle cells, to model the formation of capillaries [47,48]. This is illustrated in Figure 2. The receptors for VEGF and PDGF, i.e., VEGFR and PDGFR, have been shown to regulate cell migration through their interactions with their respective ligands [48].

The cell surface phenotype of ADSCs has been characterised as cluster of differentiation (CD)13+ CD34+ CD74+ CD90+ CD14- CD31- CD45- CD144-, suggesting that these cells are not of monocyte-macrophage lineage (CD14-, CD45-), or of endothelial lineage (CD31-, CD144), with cell surface markers subsequently varying according to the pursued pattern of differentiation. Research has further pointed out the ability of adipocyte progenitors to differentiate into white adipocytes, and of the latter to dedifferentiate into their precursor cells [49]. Furthermore, CD117 and HLA-DR (human leukocyte antigen-D related), expressed on ADSCs, are cell surface markers for adult stem cells [23,26,27,49,50].

External administration of growth factors

It has been proposed that extrinsic growth factors, such as VEGF, FGF, and platelet-rich plasma, can contribute towards an increase in fat graft survival [38,51,52]. 

In two murine models of VEGF, fat was extracted from human tissue, and following purification and isolation of human ADSCs, these were transfected with modified RNA encoded for VEGF [16], and lentiviral-vector VEGF-A, respectively [50]. In both studies, VEGF was found to correlate with increased survival of the ADSCs, individually, and of the entire fat graft. Similar results have been noted by Lu et al. [53], using adenovirus for VEGF transfection, with 74.1% graft survival, as opposed to 60.1% in untransduced ADSCs and 27.1% in controls. These studies show the potential usefulness of VEGF in fat graft survival, administered during the processing stage. 

Increased fat graft survival rates have also been reported in murine models of FGF. In a specific study, the fat survival rate in ADSCs enriched with basic FGF (βFGF) was reported at 81%, compared to ADSC alone, at 71%, and control mice, at 48%. Furthermore, western blot analysis showed an increase in VEGF and PDGF levels in these mice, in the ADSC and ADSC and bFGF groups, as compared to the control groups [44].

The addition of platelet-rich plasma (PRP) to the SVF has also shown increased fat survival, potentially due to its pro-growth-factor and pro-tissue-remodelling profile [54]. In the study of Gentile et al. [37], fat graft volume maintenance, across PRP-SVF, SVF-only, and control groups, was calculated as 69%, 63%, and 39%, at 12 months respectively, and 65%, 61%, and 30%, at 18 months, respectively. The results of a combined treatment of ADSCs with insulin and PRP were observed as early as 12 hours in the study, through the upregulation of transcription of FGF Receptor-2 (FGFR-2).

These studies, looking into VEGF, FGF, and PRP, all associated with improvement of ADSC survival, highlight the potential of their external administration, to stimulate ADSC survival and the promotion of a pro-angiogenic lineage. It should be acknowledged that although these animal models constitute progress in terms of the external administration of growth factors in ADSCs, there are substantial barriers that need to be overcome to apply these principles in clinical practice in human breast augmentation, including ethical, financial, methodological and outcome related. Other directions for further research have also been proposed, with growth factors such as insulin-like growth factor, erythropoietin, and platelet-derived growth factor [38].

A crucial concern with regards to the external administration of pro-angiogenic factors is the observed association of VEGF and FGF with cancer, as pointed out by many researchers. These, and other, growth factors have been found to be upregulated in various types of cancer, hence pushing pharmacological research towards the development of drugs against their receptors. In particular, VEGF and FGF are expressed in a variety of pathways involving endothelial cells, angiogenesis, and tissue repair, and as such, their safety profile needs to be clearly assessed, if they are to be used in cell-assisted lipotransfer in the future [55-57]. It should be emphasized that even if such growth factors are not externally administered, this does not mean that cell-assisted lipotransfer, as a novel technique, does not carry its own increased risks of cancer. These risks need to be investigated further, as short and long-term patient safety remains of utmost importance at all times.

## Conclusions

To conclude, the aim of this review has been to discuss and evaluate CAL as an alternative to AFT in breast augmentation surgery. ADSCs, extracted and isolated from the lipoaspirate, have been shown to reduce the fat resorption rates associated with AFT, yet with varying levels of success, and potentially increased complication rates. The comparison of different isolation systems has shown differing outcomes, which is a limitation of CAL in terms of large-scale use in clinical practice. Furthermore, for the time being, the legal framework of tissue culture legislation constitutes an additional barrier in the United States.

Although the cell surface and cytokine profile of ADSCs has not been fully examined yet, numerous authors have investigated and illustrated the pro-angiogenic ability of these stem cells, in both murine models and human models of breast augmentation and facial filling surgery. This pro-angiogenic ability, alongside their differentiation potential, could potentiate fat survival under conditions of post-implantation ischemia. The external addition of growth factors, such as VEGF, FGF, and PRP, may prove to be of importance in optimising fat transfer outcomes in breast augmentation surgery. It would be very interesting to explore ways in which to optimise these techniques, by comparing various modes of administration, ranging from simple injections to transfection with modified RNA plasmids, and viral vectors.

An important consideration that should be addressed is the risk of oncogenesis, as a result of the increased expression of growth factors, including VEGF and FGF. This may prove to be an important barrier in terms of the widespread use of CAL in plastic and reconstructive surgery.

To summarise the above, CAL may still be a novel technique in breast augmentation surgery, but its promising results, through the prism of multiple isolation systems, highlight the great potential for the use of this technique in clinical practice. The potential applications of CAL should be balanced with the heterogeneous results of the existing literature, and its risks, particularly that of oncogenesis. As with every surgical procedure, the primary considerations should always remain patient safety in the long term, and the optimisation of patient-reported outcomes.
